# Supplementary figures and images for: Alpha Enolase 1 Ubiquitination and Degradation Mediated by Ehrlichia chaffeensis TRP120 Disrupts Glycolytic Flux and Promotes Infection
Source: Pathogens. 2021 Jul 30;10(8):962. doi: 10.3390/pathogens10080962 (PMC8400980; doi:10.3390/pathogens10080962)

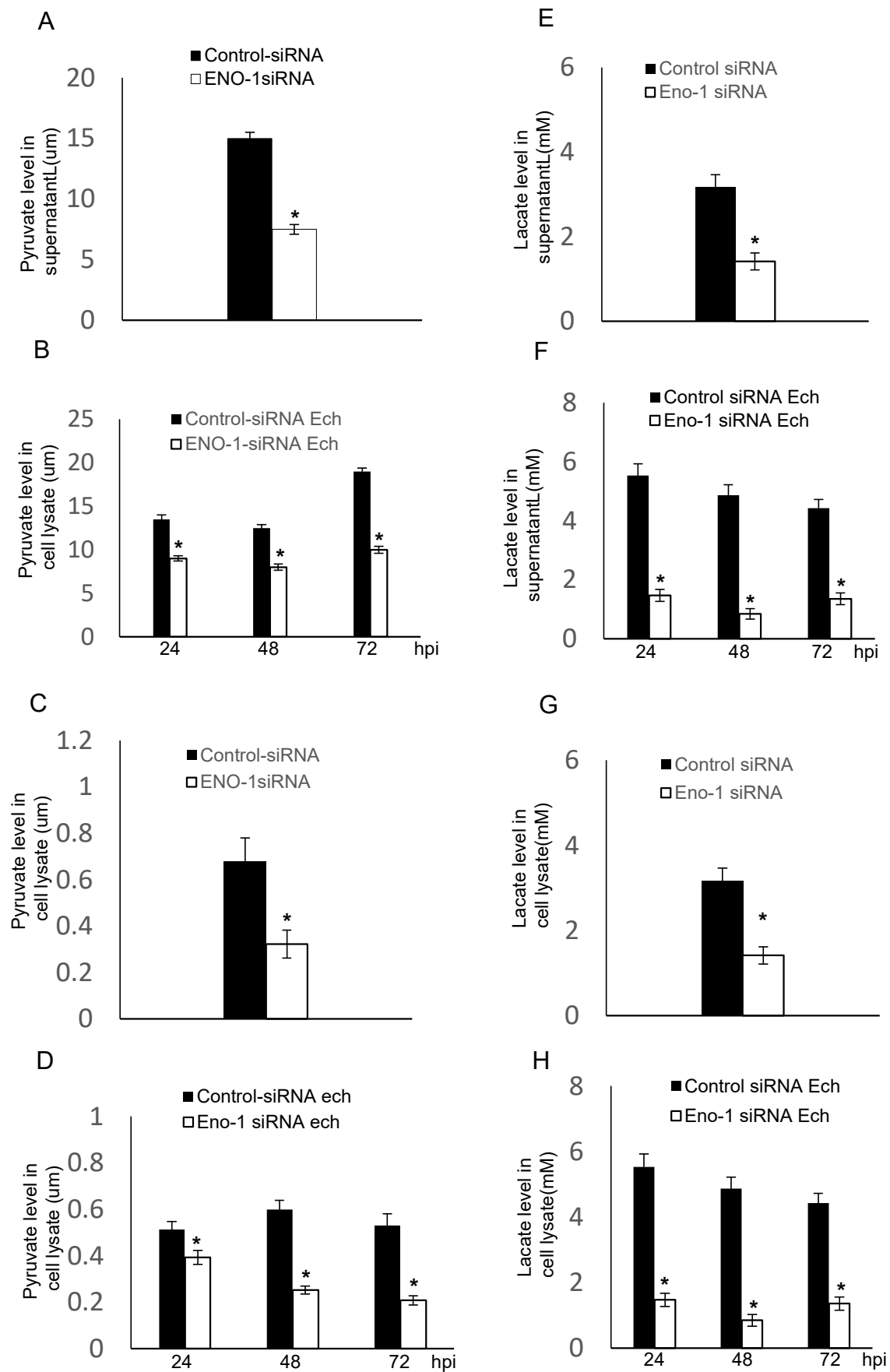

FIG S1

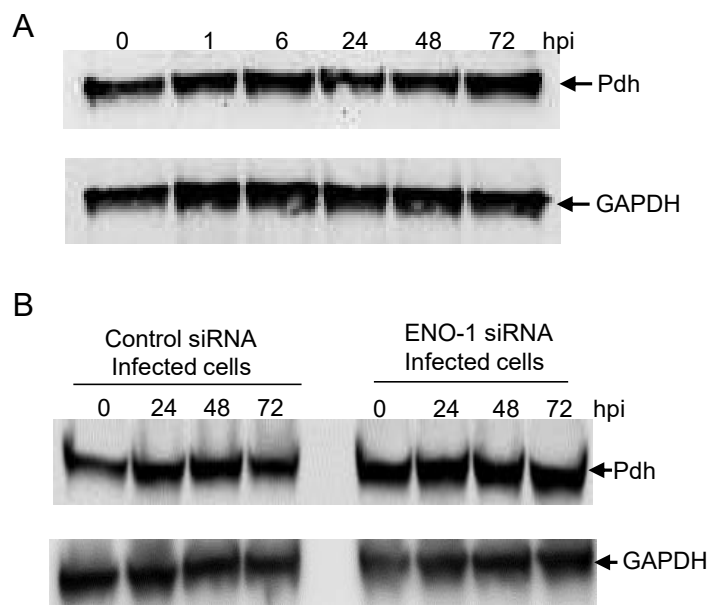

FIG S2

Supplement: Supplementary file 1 [file pathogens-10-00962-s001.zip › pathogens-1294110-supplementary.pdf]
